# Pan-drug resistant *Providencia rettgeri* contributing to a fatal case of COVID-19

**DOI:** 10.1099/jmm.0.001406

**Published:** 2021-08-27

**Authors:** Patrick Mc Gann, Matthew R. Geringer, Lindsey R. Hall, Francois Lebreton, Elizabeth Markelz, Yoon I. Kwak, Sheila Johnson, Ana C. Ong, Aubrey Powell, Tsigereda Tekle, Yehudit Bergman, Patricia J. Simner, Jason W. Bennett, Robert J. Cybulski, Brian K. White

**Affiliations:** ^1^​ Multidrug-Resistant Organism Repository and Surveillance Network (MRSN), Walter Reed Army Institute of Research, Silver Spring, Maryland, USA; ^2^​ Infectious Disease Service, Brooke Army Medical Center, San Antonio, Texas, USA; ^3^​ Department of Pathology and Area Laboratory Services, Brooke Army Medical Center, San Antonio, Texas, USA; ^4^​ Division of Medical Microbiology, Department of Pathology, Johns Hopkins University School of Medicine, Baltimore, Maryland, USA

**Keywords:** cefiderocol resistance, COVID-19, NDM carbapenemase, *Providencia rettgeri*

## Abstract

Following prolonged hospitalization that included broad-spectrum antibiotic exposure, a strain of *Providencia rettgeri* was cultured from the blood of a patient undergoing extracorporeal membrane oxygenation treatment for hypoxic respiratory failure due to COVID-19. The strain was resistant to all antimicrobials tested including the novel siderophore cephalosporin, cefiderocol. Whole genome sequencing detected ten antimicrobial resistance genes, including the metallo-β-lactamase *bla*_NDM-1_, the extended-spectrum β-lactamase *bla*_PER-1_, and the rare 16S methyltransferase *rmtB2*.

As of January 2021, 100 million people have been infected and over 2.1 million people have died worldwide [1] as a result of the novel coronavirus, severe acute respiratory syndrome coronavirus 2 (SARS-CoV-2), the etiological agent of coronavirus disease 2019 (COVID-19). Hospitalization due to COVID-19 may require prolonged use of mechanical ventilation [2], central venous catheters and extracorporeal membrane oxygenation (ECMO) [3], all of which are associated with an increased risk of infection [4].

Recent studies have shown that from 4–28 % of patients hospitalized with COVID-19 can develop secondary bacterial infections, including infections with multi-drug resistant (MDR) strains [5, 6]. Furthermore, clinical uncertainty surrounding the treatment and management of COVID-19 has resulted in extensive antimicrobial use, with a recent review showing that despite just 8 % of patients with COVID-19 developing co-infections, 72 % have received antimicrobial therapy [7]. In the absence of appropriate antimicrobial stewardship, excessive use of antimicrobials can serve as a strong selective pressure for MDR organisms [8]. In this report, we describe a pan-drug resistant (PDR) *Providencia rettgeri* cultured from the blood and a peri-rectal swab of a patient hospitalized with COVID-19 following pathogen directed therapy for a different organism.

A patient in his mid-sixties, with a history of diabetes mellitus, hypertension and an immunocompromised state presented to a hospital with hypoxic respiratory failure secondary to COVID-19. Despite a 5 day course of Remdesivir, two doses of COVID-19 convalescent plasma, and a ten-day course of dexamethasone for his infection, he was ultimately intubated on hospital day (HD) 16. He was transferred to our facility on HD 23 for veno-venous extracorporeal membrane oxygenation (VV-ECMO).

Upon transfer, he was noted to have a leukocytosis of 20.1×10^3^ cells mm^−1^ and his chest x-ray demonstrated bilateral diffuse mixed airspace opacities. He was empirically started on broad-spectrum antimicrobials (vancomycin and cefepime), which were discontinued when cultures of blood, respiratory, and urine samples had no growth. He developed a ventilator-associated pneumonia (VAP) on HD 39 and received pathogen directed therapy with tobramycin and minocycline for *Acinetobacter lwoffii *. On HD 54, he developed mixed cardiogenic and distributive shock; repeat blood cultures were obtained. Blood cultures yielded PDR *Providencia rettgeri* (MRSN 845308), identified by matrix-assisted laser desorption ionization–time of flight mass spectrometry (MALDI-TOF; Vitek MS) and antimicrobial susceptibility testing was performed using VITEK 2. A Cepheid Xpert Carba-R assay detected the *bla*_NDM_ gene. This *P. rettgeri* continued to grow from subsequent blood cultures on HD 57, 58, and 62 and was also isolated from a peri-rectal swab collected and screened for MDRO on HD 59. Without any known active antimicrobial agents according to the available antimicrobial susceptibility testing, the patient was treated with cefiderocol on HD 58. Simultaneously, the isolate was sent to the Multi-drug resistant organism Repository and Surveillance Network (MRSN) for susceptibility testing against cefiderocol and eravacycline, using Disc Diffusion (Hardy Diagnostics Cat# Z9435, 30 ug) and *E*-test (bioMérieux, Cat# 421553), respectively. In addition, the isolate was forwarded to the Medical Microbiology Department at the Johns Hopkins Hospital for broth microdilution (BMD) confirmation using the Sensititre MDRGNX2F panel (ThermoFisher). Results from both laboratories indicated that the isolate was non-susceptible to all available antimicrobials (Table 1).

**Table 1.** Antibiotic susceptibility profile of *P. rettgeri* MRSN 845308

Antibiotic	Method*	MIC (µg ml^−1^)	Interpretation†
Amikacin	Vitek 2	>=64	R
Ampicillin/Sulbactam	Vitek 2	>=32	R
Ampicillin	Vitek 2	>=32	R
Aztreonam	Vitek 2	>=64	R
Cefepime	Vitek 2	>=64	R
Cefiderocol	BMD	>32	R
Ceftazidime	Vitek 2	>=64	R
Ceftazidime/Avibactam	Vitek 2	>=16/4	R
Ceftolozane/Tazobactam	Vitek 2	>8/4	R
Ceftriaxone	Vitek 2	>=64	R
Ciprofloxacin	Vitek 2	>=4	R
Colistin	BMD	>4	R
Delafloxacin	BMD	>1	R
Eravacycline	BMD	>8	ns
Ertapenem	Vitek 2	>=8	R
Gentamicin	Vitek 2	>=16	R
Imipenem	Vitek 2	>=16	R
Imipenem/Relebactam	BMD	>16/4	R
Meropenem	Vitek 2	>=16	R
Meropenem/Vaborbactam	BMD	>16/8	R
Omadacycline	BMD	>8	R
Piperacillin/Tazobactam	Vitek 2	>=128	R
Plazomicin	BMD	>4	R
Tobramycin	Vitek 2	>=16	R
Trimethoprim/Sulfamethoxazole	Vitek 2	>=320	R

*AST testing on the Vitek2 was performed using the AST-GN95 and AST-XN09 panels. BMD, broth microdilution.

†R, resistant; S, sensitive; NS, non-susceptible. Breakpoints based on CLSI M100 Performance Standards for Antimicrobial Susceptibility Testing, 30th Edition (2020). No intermediate or resistant breakpoints have been established for eravacycline with *Enterobacterales* to date.

As recent reports have suggested that a combination of aztreonam and avibactam may be effective against metallo-β-lactamase (MBL)-producing organisms [9, 10], the patient was administered these agents on HD 65. Concurrently, *P. rettgeri* MRSN 845308 was tested for susceptibility to these agents at the MRSN using the *E*-test strip superposition method described by Emeraud and colleagues [9]. Results demonstrated a reduction in aztreonam minimum inhibitory concentration (MIC) from >256 µg ml^−1^ to 4 µg ml^−1^ (Fig. 1). The patient’s family made the decision to withdraw care secondary to multi-organ failure and overall poor prognosis and the patient passed away on HD 66.

**Fig. 1.**
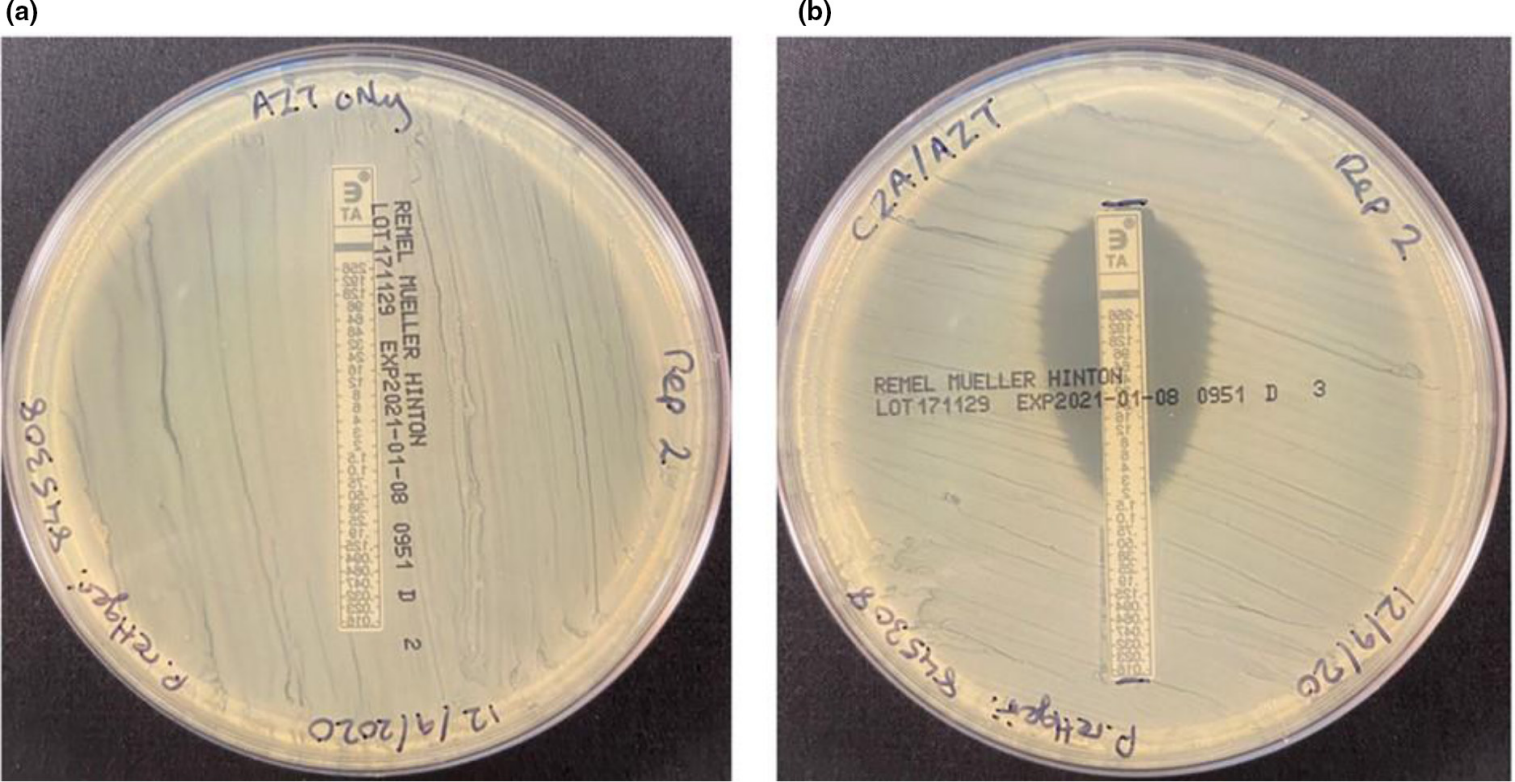
Aztreonam/Avibactam synergy testing. Aztreonam *E*-test with *Providencia rettgeri* MRSN 845308 (**a**) alone and (**b**) after an initial 10 min exposure to ceftazidime/avibactam *E*-Test using the *E*-test strip superposition method described by Emeraud and colleagues [9].

To better understand the PDR phenotype of *P. rettgeri* MRSN 845308, whole genome sequencing (WGS) was performed using an Illumina Miseq benchtop sequencer, as previously described [11]. Multiple antimicrobial resistance (AMR) genes were identified, including the MBL carbapenemase *bla*_NDM-1_, the 16S methyltransferase *rmtB2* and the extended-spectrum β-lactamase (ESBL), *bla*_PER-1_. In addition, the isolate also carried genes encoding resistance to aminoglycosides (*aac(6′)−1b, aph(3′)-Ia,* and *aadA1*), macrolides (*mph(A*)), quinolones (*qnrD1*), trimethoprim (*dfrA1*) and sulfonamides (*sul1*). Thus, the alarming antimicrobial susceptibility profile of this organism is reflected in the gene content, with the combination of PER-1 and NDM-1 contributing to the comprehensive β-lactam resistance. Similarly, the presence of the rare 16S methyltransferase RmtB2 ensures that the organism is resistant to all available aminoglycosides. When combined with the intrinsic resistance of *Providencia* to colistin and tigecycline, the resulting organism is a case study in true pan-drug resistance and constitutes a serious treatment challenge.

The high-level resistance to cefiderocol was unexpected as this agent can remain active in the presence of the most common intrinsic and acquired mechanisms of β-lactam resistance among Gram-negative bacilli, including β-lactamases and carbapenemases [12]. However, elevated MICs to this agent have been reported among Gram-negative bacilli, including *P. rettgeri *, with PER and NDM enzymes being implicated in cefiderocol resistance in strains of *Acinetobacter baumannii, Enterobacter cloacae* and *K. pneumoniae* ([13], and for a recent review see [14]). Notably, the presence of these enzymes alone may not be sufficient to confer resistance to cefiderocol [13] and multiple other resistance mechanisms involving gene mutation have also been reported [14].

*Providencia* are intrinsically resistant to the tetracyclines, and like tigecycline and omadacycline, eravacycline has poor activity against this genus. However, an early report on eravacycline indicated that this agent is two- to four-fold more active against carbapenem-resistant *Enterobacteriaceae* than tigecycline [15]. In the case of *P. rettgeri* MRSN 845308, MICs of eravacycline were >8 µg ml^−1^ by BMD and >256 µg ml^−1^ by *E*-test (data not shown). This high-level resistance is also unusual, with Livermore and colleagues reporting eravacycline MICs ≤16 µg ml^−1^ among 15 *Proteae*, including five *P *. *rettgeri* and three *P *. *stuartii* [15]. Further study on *P. rettgeri* MRSN 845308 is currently underway to unravel the mechanism of resistance to both of these agents in this strain.

The isolation of PDR *P. rettgeri* MRSN 845308 from a patient hospitalized for over 9 weeks provides a stark example of the need for comprehensive surveillance of all MDR organisms to better prevent their dissemination and inform appropriate antimicrobial stewardship. To the best of our knowledge, bacteria exhibiting this combination of antimicrobial resistance genes and antimicrobial susceptibility profile has not been described in the USA to date. Notably, the same *P. rettgeri* was also cultured from a peri-rectal swab, suggesting the patient was colonized by this strain prior to admission. Off-target antimicrobial exposure has been identified as a potential source for MDR selection and subsequent infection [16] and though speculative, it is possible that the tobramycin therapy directed against the *A. lwoffi* VAP could have resulted in off-target selection of the RmtB2-producing *P. rettgeri *, with subsequent translocation into the bloodstream. The PDR phenotype of this organism is also a cause for great concern and highlights the need for novel therapeutics. While it was promising that the organism was susceptible to the aztreonam/ceftazidime-avibactam combination *in vitro*, the efficacy of this approach could not be determined *in vivo* due to the death of the patient 1 day after treatment commenced.

Finally, Infectious Disease and Infection Prevention and Control departments should be cognizant that broad-spectrum antimicrobial treatment/prophylaxis in COVID-19 patients, combined with prolonged hospital stays, could create a favourable niche for the emergence and spread of multi-drug resistant strains and species that might otherwise be rarely associated with human disease.
